# Supplementary material for: Functional host-specific adaptation of the intestinal microbiome in hominids
Source: Nat Commun. 2024 Jan 6;15:326. doi: 10.1038/s41467-023-44636-7 (PMC10770139; doi:10.1038/s41467-023-44636-7)
Supplement: Supplementary file 1 — Supplementary Info [file 41467_2023_44636_MOESM1_ESM.pdf]

Supplementary Material

Title

Functional host-specific adaptation of the intestinal microbiome in Hominids

Authors

Rühlemann MC[1,2], Bang C[1], Gogarten JF[3,4,5,6], Hermes BM[7,8], Groussin M[1], Waschina S[9], Poyet M[8], Ulrich M[4,5], Akoua-Koffi C[10], Deschner T[11], Muyembe-Tamfum JJ[12], Robbins MM[13], Surbeck M[14,15], Wittig RM[16,17], Zuberbühler K[18,19], Baines JF[7,8], Leendertz FH[4,5], Franke A[1]

Affiliations

1 Institute of Clinical Molecular Biology, Kiel University, Kiel, Germany
2 Institute for Medical Microbiology and Hospital Epidemiology, Hannover Medical School Hannover, Germany
3 Applied Zoology and Nature Conservation, University of Greifswald, Greifswald, Germany
4 Helmholtz Institute for One Health, Greifswald, Germany
5 Epidemiology of Highly Pathogenic Microorganisms, Robert Koch Institute, Berlin, Germany
6 Viral Evolution, Robert Koch Institute, Berlin, Germany
7 Evolutionary Genomics, Max Planck Institute for Evolutionary Biology, Plön, Germany
8 Institute of Experimental Medicine, Kiel University, Kiel, Germany
9 Nutriinformatics Research Group, Institute for Human Nutrition and Food Science, Kiel University, Kiel, Germany
10 Training and Research Unit for in Medical Sciences, Alassane Ouattara University / University Teaching Hospital of Bouaké, Bouaké, Côte d'Ivoire
11 Comparative BioCognition, Institute of Cognitive Science, University of Osnabrück, Osnabrück, Germany
12 National Institute for Biomedical Research, National Laboratory of Public Health, Kinshasa, Democratic Republic of the Congo
13 Department of Primate Behavior and Evolution, Max Planck Institute for Evolutionary Anthropology, Leipzig, Germany
14 Department of Human Evolutionary Biology, Harvard University, Cambridge, Massachusetts, United States of America
15 Max Planck Institute for Evolutionary Anthropology, Leipzig, Germany
16 Institute of Cognitive Sciences, CNRS UMR5229 University Lyon 1, Bron Cedex, France
17 Taï Chimpanzee Project, CSRS, Abidjan, Côte d'Ivoire
18 Institute of Biology, University of Neuchatel, Neuchatel, Switzerland
19 School of Psychology & Neuroscience, University of St Andrews, St Andrews, Scotland (UK)

Supplementary Figures

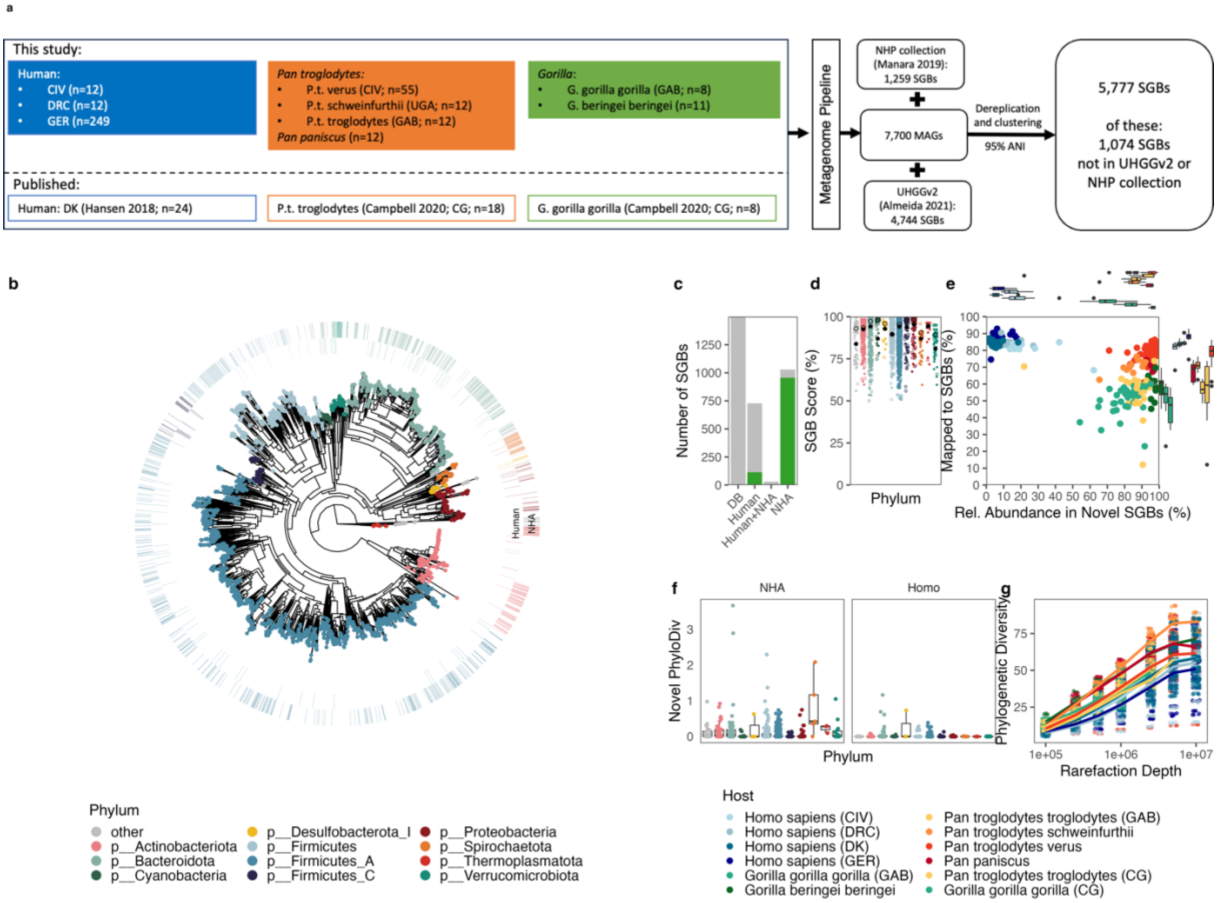

Supplementary Figure S1: Genome statistics of newly reconstructed MAGs and representative MAGs from UHGGv2 and Manara et al. (2019). (a) overview on included datasets and data processing (b) Phylogeny of all 2,943 SGB representative MAGs based on 120 bacterial and 53 archaeal universal single-copy marker genes. (c) SGB origin and novelty (green) based on reconstructed MAG sequences from human and NHP hosts, as well as reference databases (UHGGv2 and Manara et al. 2019). (d) MAGScoT scores of all SGB representative genome sequences. Filled shapes represent previously recovered SGBs, filled shapes represent novel SGBs. (e) Mapping success and relative abundance in novel SGBs for the different host groups (n=211). (f) overall novelty by NHP and Human host groups measured by Faith's phylogenetic diversity. (g) per-sample and per-group phylogenetic diversity in relation to metagenomic rarefaction depth based on n=211 independent samples (n_{Human}=71, $n_{Chimpanzee}$=91, n_{Bonobo}=12, $n_{Gorilla}$=40)

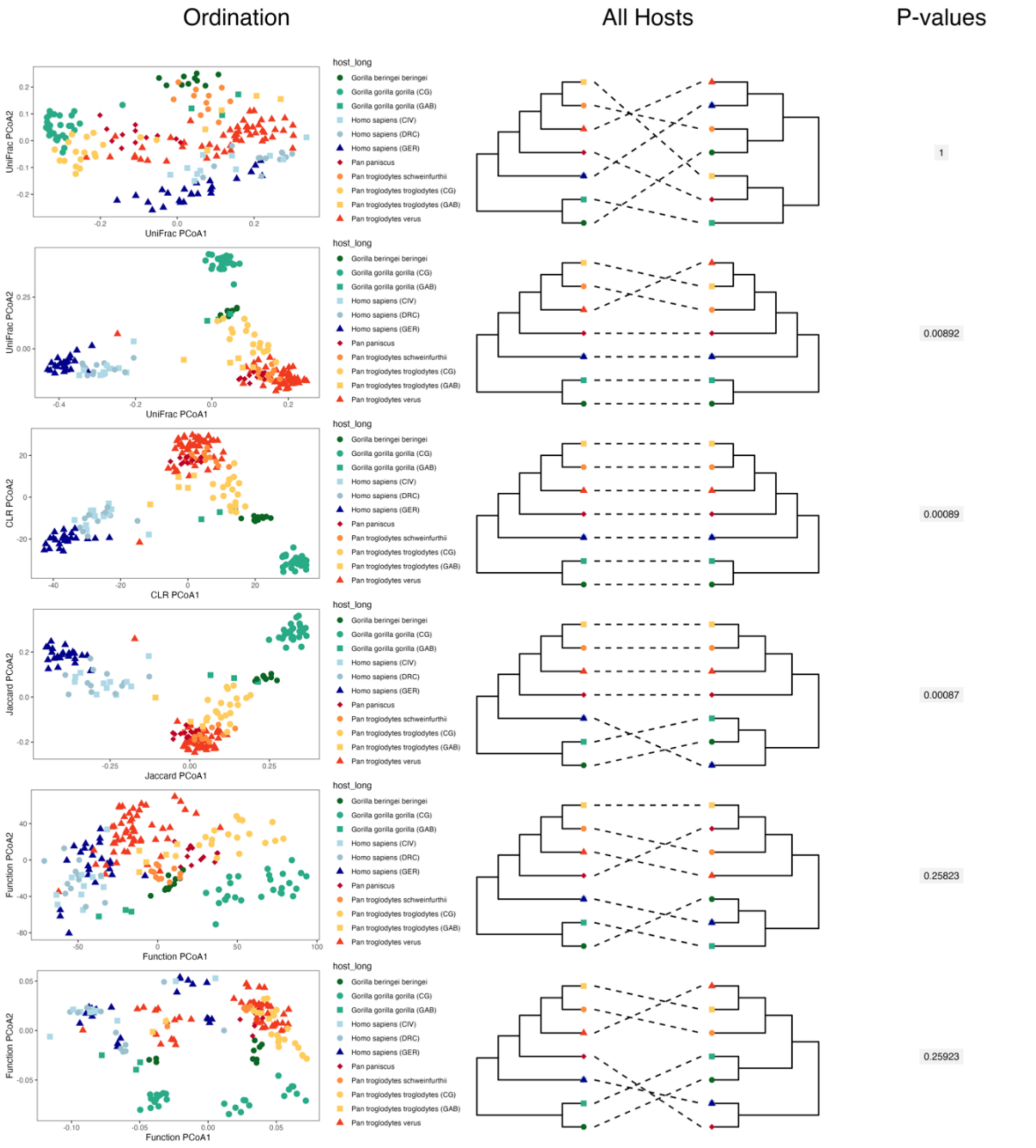

Supplementary Figure S2: Ordination, hierarchical clustering and results from the phylosymbiosis analysis for six distance measures: weighted UniFrac, unweighted UniFrac, genus-level Aitchison distance (CLR-transformed genus abundances), genus-level Jaccard distance, as well as distances based on KEGG ontology functional abundances and presence/absence patterns of KEGG ontology functions.
