# Supplementary material for: Functional host-specific adaptation of the intestinal microbiome in hominids
Source: Nat Commun. 2024 Jan 6;15:326. doi: 10.1038/s41467-023-44636-7 (PMC10770139; doi:10.1038/s41467-023-44636-7)
Supplement: Supplementary file 3 — Description of Additional Supplementary Files [file 41467_2023_44636_MOESM3_ESM.docx]

**Supplementary Data**

All supplementary data are found in the supplied excel file

**Supplementary Data legends**

File Name: Supplementary Data 1

Description: Sample Overview

File Name: Supplementary Data 2

Description: All metagenome-assembled genomes and representatives from the UHGGv2 and Manara et al.; including genome score (Magscot score), taxonomic annotation

File Name: Supplementary Data 3

Description: Abundances of all SGBs across the human and African great ape samples

File Name: Supplementary Data 4

Description: P-values from the phylosymbiosis analysis

File Name: Supplementary Data 5

Description: Results from the analysis of group-specific enrichments for microbial genera

File Name: Supplementary Data 6

Description: Results from the analysis of group-specific enrichments for microbial functions

File Name: Supplementary Data 7

Description: Results from the meta-analysis of group-specific enrichments for KEGG annotations

File Name: Supplementary Data 8

Description: Family level results of enrichment analysis of microbial functions differing between humans living in Europe or Africa

File Name: Supplementary Data 9

Description: Meta-analysis results of enrichment analysis of microbial functions differing between humans living in Europe or Africa

File Name: Supplementary Data 10

Description: Enrichment analysis of KEGG annotations differing between humans living in Europe or Africa

File Name: Supplementary Data 11

Description: Results of the enrichment analysis of microbial functions (KOs) differing between NHAs and humans within microbial genera

File Name: Supplementary Data 12

Description: Meta-analysis of the enrichment analysis of microbial functions (KOs) differing between NHAs and humans within microbial genera

File Name: Supplementary Data 13

Description: Results of the DTL tree reconciliation analsis of 752 single-copy genes in Prevotella

File Name: Supplementary Data 14

Description: Results of the co-phylogeny analysis of 209 microbial subtrees

File Name: Supplementary Data 15

Description: Enrichment and depletion of cophylogeny signals for 26 microbial families with SGBs included in the cophylogeny analysis

File Name: Supplementary Data 16

Description: Information on genome size, gene count and 62 in silico inferred traits for 1017 SGBs included in the cophylogeny analysis

File Name: Supplementary Data 17

Description: Results for the 45 traits subjected to the phylogenetic mixed model analysis
