# Supplementary material for: Functional host-specific adaptation of the intestinal microbiome in hominids
Source: Nat Commun. 2024 Jan 6;15:326. doi: 10.1038/s41467-023-44636-7 (PMC10770139; doi:10.1038/s41467-023-44636-7)
Supplement: Supplementary file 5 — Reporting Summary [file 41467_2023_44636_MOESM5_ESM.pdf]

nature portfolio

Corresponding author(s): Malte C Ruehlemann

Last updated by author(s): Dec 13, 2023

Reporting Summary

Nature Portfolio wishes to improve the reproducibility of the work that we publish. This form provides structure for consistency and transparency in reporting. For further information on Nature Portfolio policies, see our Editorial Policies and the Editorial Policy Checklist.

Statistics

For all statistical analyses, confirm that the following items are present in the figure legend, table legend, main text, or Methods section.

n/a	Confirmed	
☐	☒	The exact sample size (*n*) for each experimental group/condition, given as a discrete number and unit of measurement
☐	☒	A statement on whether measurements were taken from distinct samples or whether the same sample was measured repeatedly
☐	☒	The statistical test(s) used AND whether they are one- or two-sided<br>*Only common tests should be described solely by name; describe more complex techniques in the Methods section.*
☐	☒	A description of all covariates tested
☐	☒	A description of any assumptions or corrections, such as tests of normality and adjustment for multiple comparisons
☐	☒	A full description of the statistical parameters including central tendency (e.g. means) or other basic estimates (e.g. regression coefficient) AND variation (e.g. standard deviation) or associated estimates of uncertainty (e.g. confidence intervals)
☐	☒	For null hypothesis testing, the test statistic (e.g. *F*, *t*, *r*) with confidence intervals, effect sizes, degrees of freedom and *P* value noted<br>*Give P values as exact values whenever suitable.*
☒	☐	For Bayesian analysis, information on the choice of priors and Markov chain Monte Carlo settings
☒	☐	For hierarchical and complex designs, identification of the appropriate level for tests and full reporting of outcomes
☐	☒	Estimates of effect sizes (e.g. Cohen's *d*, Pearson's *r*), indicating how they were calculated

Our web collection on statistics for biologists contains articles on many of the points above.

Software and code

Policy information about availability of computer code

Data collection	No software was used
Data analysis	As also stated in the manuscript, all code to process sequencing files to generate the presented results and manuscript figures is available via https://github.com/mruehlemann/greatapes_mgx_scripts. Software used: R (4.2); BBmap (38.57); Spades (3.15.5); Minimap (2.24-r1122); Samtools (1.9); HMMER (3.3.2); Metabat (2.15); vamb (3.0.9); Maxbin (2.2.4); concoct (1.1.0); MAGScoT (1.0); GNU parallel (20140922); PRODIGAL (2.6.3); dRep (3.4.0); GTDBtk (2.1.0); emapper (2.1.3); salmon (1.9.0); mmseqs2 (13.45111); Ranger-DTL (v.2.0); R packages: tidyverse (1.3.2), TreeDist (2.5.0), phangorn (2.10.0), picante (1.8.2), vegan (2.6-4), ggtree (3.6.2)

For manuscripts utilizing custom algorithms or software that are central to the research but not yet described in published literature, software must be made available to editors and reviewers. We strongly encourage code deposition in a community repository (e.g. GitHub). See the Nature Portfolio guidelines for submitting code & software for further information.

Data

Policy information about availability of data

All manuscripts must include a data availability statement. This statement should provide the following information, where applicable:

- Accession codes, unique identifiers, or web links for publicly available datasets
- A description of any restrictions on data availability
- For clinical datasets or third party data, please ensure that the statement adheres to our policy

All metagenomic sequencing data is available via the NCBI BioProject accession IDs PRJNA692042, PRJNA539933 and PRJNA491335. Genome taxonomy database

version 207v2, eggNOG database (v5.0)

Research involving human participants, their data, or biological material

Policy information about studies with human participants or human data. See also policy information about sex, gender (identity/presentation), and sexual orientation and race, ethnicity and racism.

Reporting on sex and gender	We did not stratify or correct for sex or gender effects in the analysis. Our analyses focus on the comparison of gut metagenomes from either distinct hominid species or between human subgroups from populations with differences in human development index. The effects of sex and/or gender are negligible in this context.
Reporting on race, ethnicity, or other socially relevant groupings	In our analysis we included fecal samples from humans from Germany and two rural locations in Africa (Côte d'Ivoire and Democratic Republic of the Congo). These samples represent a spectrum of human populations with differences in Human Development Index and associated factors. Due to the rather low sample sizes (24 GER, 24 DK, 12 CIV, 12 DRC), comparisons were made only based on geographic location (EUR vs. AFR) without further stratifying / subgrouping the samples.
Population characteristics	Raw metagenomic data were previously published: https://www.pnas.org/doi/10.1073/pnas.2013535118 and https://pubmed.ncbi.nlm.nih.gov/32203121/. Individuals were randomly selected from larger-scale sample collections, , excluding individuals with known chronic inflammatory conditions and/or antibiotics use 12 weeks prior to sampling. No additional covariates were considered in the selection of individuals and the analysis
Recruitment	Samples from Germany were randomly selected from population-level cohorts, excluding individuals with known chronic inflammatory conditions and/or antibiotics use 12 weeks prior to sampling. Samples from individuals from CIV and DRC were collected as part of local health projects. Raw metagenomic data were previously published: https://www.pnas.org/doi/10.1073/pnas.2013535118 and https://pubmed.ncbi.nlm.nih.gov/32203121/
Ethics oversight	Ethical approval for work on human samples was obtained from the Local Ethics Committee Germany, Kiel (reference number A156/03), the Ivorian ethics commission (Comité national d'éthique et de la recherche [CNER], permit number 101 10/MSHP/CNER/P) and the Congolese ethics commission (Comité d'Éthique, Ministère de l'Enseignement Supérieur et Universiaire, permit number ESO/CE/018/11). All procedures performed in studies involving human participants were in accordance with the ethical standards of the institutional and/or national research committee and with the 1975 Helsinki declaration and its later amendments or comparable ethical standards. Sampling of wild-living great apes and human populations in Africa were granted by: Bwindi Impenetrable Forest National Park, Uganda (Gorilla beringei beringi, Pan troglodytes schweinfurthii): the Uganda National Council for Science and Technology and the Uganda Wildlife Authority; Kokolopori Bonobo Reserve and Bandundu region, Democratic Republic of the Congo (Pan paniscus, Human): the Ministere de Recherche Scientifique et Technologie, Democratic Republic of the Congo; Loango National Park, Gabon (Gorilla gorilla gorilla, Pan troglodytes troglodytes): the Agence Nationale des Parcs Nationaux, the Centre National de la Recherche Scientifique et Technique of Gabon; Taï National Park and region, Côte d'Ivoire (Pan troglodytes verus, Human): the Ministère de l'Enseignement Supérieur et de la Recherche Scientifique, the Ministère des Eaux et Fôrets in Côte d'Ivoire, and the Office Ivoirien des Parcs et Réserves. Local researchers from CIV and DRC contributing to the conducted research and fulfilling the authorship criteria were included as co-authors.

Note that full information on the approval of the study protocol must also be provided in the manuscript.

Field-specific reporting

Please select the one below that is the best fit for your research. If you are not sure, read the appropriate sections before making your selection.

☐ Life sciences ☐ Behavioural & social sciences ☒ Ecological, evolutionary & environmental sciences

For a reference copy of the document with all sections, see nature.com/documents/nr-reporting-summary-flat.pdf

Ecological, evolutionary & environmental sciences study design

All studies must disclose on these points even when the disclosure is negative.

Study description	Fecal samples were collected from humans ((n=48) and African great apes, including two gorilla subspecies (Gorilla gorilla gorilla, Gabon (GAB), n=8; Gorilla beringei beringei, Uganda (UGA), n=11), three chimpanzee subspecies (Pan troglodytes verus, CIV, n=55; P.t. troglodytes, GAB, n=11; P.t. schweinfurthii, UGA, n=12), and bonobos (Pan paniscus, DRC, n=12). Additional publicly available data from G.g. gorilla (n=28) and P.t. troglodytes (n=18) from Republic of Congo (CG) were included. Additional publicly available data from humans (n=24) from Denmark (DK) were included. The trial was purely observational, no treatments were performed. All samples included were from individual humans and great apes.
Research sample	Host were chosen based on the availability from participating research institutions and aimed to cover a broad range of African great ape (sub-species) including two gorilla subspecies (Gorilla gorilla gorilla, Gabon (GAB), n=8; Gorilla beringei beringei, Uganda (UGA), n=11), three chimpanzee subspecies (Pan troglodytes verus, CIV, n=55; P.t. troglodytes, GAB, n=11; P.t. schweinfurthii, UGA, n=12), and bonobos (Pan paniscus, DRC, n=12). Great ape groups were habituated and not actively interacted with.

Sampling strategy	Sample sizes were chosen to meet a common ground between broadness of included hosts, statistical power and available funds. Many of the included host species / groups were never included in fecal metagenomic studies before, thus the choice of 10-12 individuals per group was chosen appropriate.
Data collection	Samples from wild-living African great apes were collected from habituated groups after defecation. Data on feces-to-individual mappings were recorded in the field and later recorded electronically.
Timing and spatial scale	Single-timepoint data was used for all included individuals. Sampling periods: Gorilla beringei beringei from Bwindi: 2011; Gorilla gorilla gorilla from Loango: 2015-2016; Pan paniscus from Kokolopori: 2017; Pan troglodytes troglodytes from Loango: 2016-2017; Pan troglodytes schweinfurthii from Budongo: 2004-2008: Pan troglodytes verus from Taï National Park: 2001-2014; humans in Democratic Republic of Congo: 2011; humans in Côte d'Ivoire: 2011; humans in Germany: 2012-2014
Data exclusions	12 samples of each African great ape subgroup were subjected to shotgun metagenomic sequencing, except for the P.t. verus group for which 55 samples were included. Four G. b. beringei, one G.g. gorilla and one P.t. troglodytes sample failed sequencing, not producing any data. Further samples with less than 1 Million mapped reads were removed from further analyses. This resulted in the removal of samples from the analysis for the groups G.b. beringei (n=1), G.g. gorilla (nGAB=5, nCG=1), P.t. troglodytes (nGAB=4), P.t. schweinfurthii (n=1) and humans from CIV (n=1)
Reproducibility	Single-timepoint data was used for all included individuals. By collecting samples from multiple individuals from each subgroup we can confidently identify group-specific robust signatures in the composition of the fecal microbiota. All information ond data, software and code used for the processing and dowstream analysis are provided in public repositories on the projects github page for reproducibility.
Randomization	Not applicable as sampling was performed from populations and not treatments were applied
Blinding	Not applicable as sampling was performed from populations and not treatments were applied

Did the study involve field work? ☒ Yes ☐ No

Field work, collection and transport

Field conditions	Samples were collected from multiple locations including cities, rural villages and rain forests. Sampling periods: Gorilla beringei beringei from Bwindi: 2011; Gorilla gorilla gorilla from Loango: 2015-2016; Pan paniscus from Kokolopori: 2017; Pan troglodytes troglodytes from Loango: 2016-2017; Pan troglodytes schweinfurthii from Budongo: 2004-2008: Pan troglodytes verus from Taï National Park: 2001-2014; humans in Democratic Republic of Congo: 2011; humans in Côte d'Ivoire: 2011; humans in Germany: 2012-2014
Location	Bwindi National Park, Uganda; Kokolopori Bonobo Reserve, Democratic Republic of the Congo; Loango National Park, Gabon; Taï National Park, Côte d'Ivoire
Access & import/export	Bwindi—The mountain gorilla survey was conducted by the Uganda Wildlife Authority, l'Institut Congolais pour la Conservation de la Nature, the Rwanda Development Board, the International Gorilla Conservation Programme, the Max Planck Institute for Evolutionary Anthropology, Conservation Through Public Health, the Mountain Gorilla Veterinary Project, the Institute for Tropical Forest Conservation, and The Dian Fossey Gorilla Fund and was conducted in compliance with the regulations of and permission of the Uganda National Council for Science and Technology and the Uganda Wildlife Authority; Kokolopori—permission was granted through the Ministere de Recherche Scientifique et Technologie, Democratic Republic of the Congo, and work was supported by the Vie Sauvage, the Bonobo Conservation Initiative; Loango—permission was granted by the Agence Nationale des Parcs Nationaux, the Centre National de la Recherche Scientifique et Technique of Gabon; Taï National Park—permission was granted by the Ministère de l'Enseignement Supérieur et de la Recherche Scientifique, the Ministère des Eaux et Fôrets in Côte d'Ivoire, and the Office Ivoirien des Parcs et Réserves, and work was supported by the Centre Suisse de Recherches Scientifiques en Côte d'Ivoire and the staff members of the Taï Chimpanzee Project.
Disturbance	No disturbances affecting the studies results were recorded

Reporting for specific materials, systems and methods

We require information from authors about some types of materials, experimental systems and methods used in many studies. Here, indicate whether each material, system or method listed is relevant to your study. If you are not sure if a list item applies to your research, read the appropriate section before selecting a response.

Materials & experimental systems

n/a	Involved in the study
☒	☐ Antibodies
☒	☐ Eukaryotic cell lines
☒	☐ Palaeontology and archaeology
☐	☒ Animals and other organisms
☒	☐ Clinical data
☒	☐ Dual use research of concern
☒	☐ Plants

Methods

n/a	Involved in the study
☒	☐ ChIP-seq
☒	☐ Flow cytometry
☒	☐ MRI-based neuroimaging

Animals and other research organisms

Policy information about studies involving animals; ARRIVE guidelines recommended for reporting animal research, and Sex and Gender in Research

Laboratory animals	No laboratory animals were included in the study
Wild animals	Feces from wild-living, habituated animals were collected after defecation without interfering with the individual animals. Host species included two gorilla subspecies (Gorilla gorilla gorilla, Gabon (GAB), n=8; Gorilla beringei beringei, Uganda (UGA), n=11), three chimpanzee subspecies (Pan troglodytes verus, CIV, n=55; P.t. troglodytes, GAB, n=11; P.t. schweinfurthii, UGA, n=12), and bonobos (Pan paniscus, DRC, n=12).
Reporting on sex	We did not stratify or correct for sex or gender effects in the analysis. Our analyses focus on the comparison of gut metagenomes from either distinct hominid species or between human subgroups from populations with differences in human development index. The effects of sex and/or gender are negligible in this context.
Field-collected samples	Fecal samples were collected immediately after defecation, and, depending on the local infrastructure, either stored in RNAlater and frozen at -20°C or stored in a cryotube, cooled in a thermos until return to the field laboratory, and subsequently snap frozen in liquid nitrogen.
Ethics oversight	Appropriate government permits and permission to conduct research on wild primates were granted by the relevant authorities. Bwindi—The mountain gorilla survey was conducted by the Uganda Wildlife Authority, l'Institut Congolais pour la Conservation de la Nature, the Rwanda Development Board, the International Gorilla Conservation Programme, the Max Planck Institute for Evolutionary Anthropology, Conservation Through Public Health, the Mountain Gorilla Veterinary Project, the Institute for Tropical Forest Conservation, and The Dian Fossey Gorilla Fund and was conducted in compliance with the regulations of and permission of the Uganda National Council for Science and Technology and the Uganda Wildlife Authority; Kokolopori—permission was granted through the Ministere de Recherche Scientifique et Technologie, Democratic Republic of the Congo; Loango—permission was granted by the Agence Nationale des Parcs Nationaux, the Centre National de la Recherche Scientifique et Technique of Gabon; Taï National Park—permission was granted by the Ministère de l'Enseignement Supérieur et de la Recherche Scientifique, the Ministère des Eaux et Fôrets in Côte d'Ivoire, and the Office Ivoirien des Parcs et Réserves

Note that full information on the approval of the study protocol must also be provided in the manuscript.
